# Recapitulating the Tumor Ecosystem Along the Metastatic Cascade Using 3D Culture Models

**DOI:** 10.3389/fonc.2015.00170

**Published:** 2015-07-29

**Authors:** Jiyun Kim, Kandice Tanner

**Affiliations:** ^1^Laboratory of Cell Biology, Center for Cancer Research, National Cancer Institute, National Institutes of Health, Bethesda, MD, USA; ^2^Nano System Institute, Seoul National University, Seoul, South Korea

**Keywords:** tumor microenvironment, 3D culture models, ecology, biomaterials and nanotechnology, hydrogels

## Abstract

Advances in cancer research have shown that a tumor can be likened to a foreign species that disrupts delicately balanced ecological interactions, compromising the survival of normal tissue ecosystems. In efforts to mitigate tumor expansion and metastasis, experimental approaches from ecology are becoming more frequently and successfully applied by researchers from diverse disciplines to reverse engineer and re-engineer biological systems in order to normalize the tumor ecosystem. We present a review on the use of 3D biomimetic platforms to recapitulate biotic and abiotic components of the tumor ecosystem, in efforts to delineate the underlying mechanisms that drive evolution of tumor heterogeneity, tumor dissemination, and acquisition of drug resistance.

## Introduction

Patient survival following the diagnosis of certain types of cancers has significantly improved due to our current understanding of the molecular basis of tumor etiology (1–4). Tissue biopsies remain the first line of diagnosis for solid cancers (5). Histopathology provides physicians with local genetic and epigenetic information that guides the choice of an appropriate therapeutic regimen (5). Unfortunately, this initial intervention against a single molecular pathway or a specific mutant type may not be an effective way to treat the tumor as a whole or emerging phenotypes. Delivering targeted therapy that is consistently effective as the tumor evolves remains challenging (6, 7). One limitation may be due to the fact that a biopsy may not be representative of the larger tumor mass or is not predictive of a distant lesion in the event of metastatic disease. The other is that treatment in itself may select for tumor cells harboring less than desirable traits that represented a small fraction of cells in the original tumor (6, 7). Intratumoral heterogeneity is thought to allow for rapid adaptation to external stress, selecting for emergent phenotypes that can adapt to the foreign environments of distal organs and are drug-resistant (8). Regrettably, intratumoral heterogeneity persists after tumor cells have left the primary site and disseminated to establish metastatic lesions. Although many disseminated tumor cells (DTCs) circulate within the bloodstream, only a small subset of these will actually form a lesion. Ostensibly, many do not survive environmental pressures experienced during hematogenous or lymphatic spread (9). A subset of those that establish metastatic lesions may remain dormant for periods of time ranging from several months to decades (9) These quiescent DTCs may then awaken due to perturbations of the microenvironment (10–12) leading to tumor recurrence with inter- and intra-tumoral heterogeneity, largely resistant to drug intervention. Therefore, selecting for cells that either do not survive after dissemination or remain quiescent are possible areas for therapeutic intervention to prevent metastasis and increase treatment efficacy (9, 13, 14).

For some hematological cancers, such as acute myeloid leukemia, cancer stem cells (CSCs) or tumor-initiating cells are thought to drive the observed heterogeneities (15, 16). CSCs employ mechanisms such as self-renewal and differentiation into multiple tumor cell types to drive tumor growth, causing relapse, metastasis, and sometimes even recovery following therapeutic intervention (15, 17). Thus, CSCs increase the adaptive capability as they drive the evolution of distinct cancer cell populations to shape the tumor ecosystem. In such cases, targeting and removing the CSC population may sufficiently suppress the evolution of phenotypic and genotypic heterogeneities. Cairns and Nowell provided conceptual models that allow these observed tumor heterogeneities to be described by Darwinian principles (18, 19).

However, in most solid cancers, the evolution of heterogeneity is largely stochastic (20). The resultant genomic instability (21) and epigenetic changes are driven by heterotypic cell interactions within a dynamic extracellular milieu in response to external perturbations such as changes in oxygen tension, stiffness, or nutrient, and pH gradients (22). Diversity arises due to proliferation of sub-clones that survive selective pressures and environmental perturbations such as hypoxia, drug treatment, and reduced nutrients and evolve to increase phenotypic and functional heterogeneity within the tumor (22–24). Simply put, intratumoral heterogeneity does not merely develop from a random collection of mutated cells with uniform levels of proliferation (25). It also stems from interactions with the biotic (biochemical) and abiotic (physical) components of the environment. Drawing from lessons learned in the field of ecology, genetically reduced populations are more vulnerable to disease outbreaks, other environmental stresses (26), and the accumulation of deleterious mutations (27). So how do we design a therapy to reduce tumor heterogeneity?

The microenvironment is emerging as a critical factor in malignant progression, metastasis, and tumor etiology (20, 28, 29). The physical properties of the microenvironment, including the stiffness, dimension, and topography, work in concert with biochemical signals to have profound impacts on cell fate, tissue assembly, and malignancy (30, 31). A dynamic tumor microenvironment may not only contribute to systemic metastasis, but also significantly modify drug efficacy (31–33). These dynamic, bi-directional interactions between individual tumor cells and collectively with other cell types and with the extracellular matrix (ECM) milieu can be likened to a dynamic ecosystem (22, 34–36). In nature, an introduced foreign species or an aberrant member may disrupt the delicately balanced interactions that normally exist in an ecosystem between members of a species, predator and prey, animals and plants, or between life and the abiotic environment, compromising survival of other components, and perhaps even leading to the collapse of the entire system. Similarly, tumor cells detrimentally transform the balanced interactions within the surrounding normal tissue (e.g., between clonal subpopulations, immune cells and their targets, epithelial and stromal cells, or between cells and the extracellular microenvironment) to achieve and maintain tumor homeostasis (34, 35). Cancer can be likened to a disease state in which a new species evolves, eventually dominating the original organ. In many ways, interactions between tumor cells and their microenvironment mimic those observed during normal organogenesis. The concept of the tumor as an abnormal organ has been rigorously reviewed elsewhere (34, 35). Here, we build on this concept to understand the tumor dynamics of the transition from normal to malignant, from malignant to metastatic, and the meta-states that arise following treatment, in terms of the interactions between tumor cells and their microenvironment. One approach may be to model the evolutionary response of the tumor from an ecological perspective, so that a treatment would mitigate or undermine tumor heterogeneity. But first, we must understand the creation and evolution of the tumor microenvironment.

A recent review by Pienta and colleagues presents the argument for ecological therapy and consideration of the microenvironment as a possible approach to designing novel cancer therapeutics (37). An additional advantage of an ecological approach is the potential to exploit the existing mathematical and computational framework based on Darwinian dynamics that model the tumor heterogeneities in the context of microenvironmental regulation (20, 38). Increased efficacy of disease management may be achieved by combining treatments such as surgery, chemotherapy, or radiotherapy with approaches that are capable of reducing tumor diversity and “healing” the microenvironment (31, 39). Therefore, deciphering the underlying mechanisms that govern the establishment of the physico-chemical properties of the tumor-promoting niche is desirable. Continuing with our analogies drawn from the field of ecology, the concept of ecosystem engineering is the process whereby organisms modify (directly or indirectly) resources available to other species by causing physical state changes in biotic and abiotic components (40). Consequently, habitats are modified, synthesized, or maintained. Ecosystem engineers change environments by altering their physical structures (40). One example of ecosystem engineering, also referred to as niche construction (41), is dam creation by beavers (40). They physically remodel and destroy wooded areas, with profound effects on both soil drainage and water allocation (40). Another example is how earthworms chemically and physically modify soil as they burrow (40). Similarly, tumor cells work in concert with stromal cells to physically remodel and chemically define their environment, which then drives further tumor phenotypic evolution (42). So how do we recapitulate tumor diversification and the dynamics of physico-chemical microenvironment *in vitro*?

Three-dimensional (3D) culture systems in which extracellular matrices that biochemically and physically mimic the *in vivo* composition of organs have been extensively used to study normal epithelial organogenesis for the breast, prostate, salivary gland, and kidney (43–45). In addition, pharmaceutical studies involving endothelial cell-derived 3D spheroids have been extensively used for evaluating the pro- and anti-angiogenic potential of drugs targeting the tumor vasculature (46). In these biomimetic systems, cells adopt physiological morphologies and the appropriate cell signaling is achieved. 3D culture models can also be used to recapitulate the abiotic and biotic components of the tumor ecosystem. Namely, the abiotic components such as the spatio-temporal gradients of chemicals, oxygen tension, and mechanical cues can be robustly engineered using applications from microfluidics, electrospinning, and soft lithography (47, 48). The biotic components such as the ECM environment and heterotypic cell interactions can also be approximated using biomimetic platforms and co-culture systems. Although the conventional two-dimensional (2D) tissue culture system has contributed enormously to the progress of cancer biology, cancer cells encounter diverse 3D topographies and architectures *in vivo*. Thus, 3D culture models allow for deconstructing the complexity of cancer by recapitulating emergent, population-level characteristics of the tumor microenvironment.

## Tumor Cells as Ecosystem Engineers – Defining the Tumor ECM Niche

A hallmark of malignant transformation is the loss of anatomical organization (49, 50). Tumor cells aberrantly proliferate, remodel, and rebuild a new microenvironment by releasing extracellular signaling molecules that promote tumor angiogenesis and ECM remodeling (34, 35, 42, 51). In a seminal paper, Dvorak postulated that tumors were simply “wounds that did not heal.” He reasoned that common cellular and molecular mechanisms are activated in wounds and cancerous tissue based on the observation that tumor stroma bore a strong similarity to the observed granulation tissue in skin wounds (52). However, the direct link between wound repair, chronic inflammation, and cancer was observed in later experiments where tumors were induced by the Rous sarcoma virus (a potent uncovers) only at the site of injections in newly hatched chickens but not *in ovo* (53). Wounding in an infected animal at the site of injury induced additional tumors away from the site of injection (53). Treatment with anti-inflammatory therapy prevented tumor formation, thus showing the effect of inflammation on tumorigenesis (53, 54). These observations highlight the importance of dynamic interplay between the tumor and the inflammatory microenvironment and have been reviewed extensively elsewhere (55–57). Here, we focus specifically on the ECM milieu in niche construction. The chemistry and physical properties of the ECM is dynamically tuned during *de novo* remodeling of the tumor microenvironment (42, 58). An overabundance of diverse ECM proteins and ECM remodeling enzymes is found in solid cancers (42, 51, 59–62). In addition to these chemical changes, physical properties of the tumors are altered. For example, tumors are often stiffer to the touch than the adjacent normal tissue (30, 63). In breast cancer, the fibrillar architecture of type I collagen surrounding the tumor is highly linearized as compared to normal tissue, which is thought to facilitate invasion into neighboring tissue (63, 64). These changes in ECM composition and architecture potentiate tumor-promoting changes in various signaling pathways (42). Specifically, perturbations in ECM synthesis, degradation, density, and rigidity promote cancer cell proliferation, migration, and invasion, and modulate inflammatory responses and lymphangiogenesis (35). The resulting abnormal microenvironment can exert selective pressure on cancer cell populations, increasing genomic instability and population diversity (42, 65).

## Ecosystem Engineering during Metastatic Colonization – Defining the Metastatic ECM Niche

Disseminated tumor cells leave the original tumor to initiate the metastatic cascade (66). After successful navigation of the circulatory system, a subset of these DTCs then exits via capillaries at a distant site and infiltrates the tissue (66). These DTCs colonize their new environment by poorly understood mechanisms involving adherence, remodeling, and proliferation. The term “colonization” here defines the establishment of the tumor niche, net tumor cell proliferation, and angiogenesis in the formation of a metastatic lesion. In 1889, Paget hypothesized that the interaction between the tumor cells, the “seeds” and the host environment, the “soil,” determines metastatic outcome (67). This hypothesis predicted that the tissue-specific biological and biochemical conditions (defined by the resident cell populations, extracellular matrices, and vasculature) might selectively facilitate tumor metastasis, explaining the organ selectivity of certain metastatic cancers. It is now well appreciated that the continuous dynamic and reciprocal relationship between cells and their microenvironment in which the mechanical properties of tissue including the geometry, topography, and elasticity of the ECM can provide intrinsic signals to cells that have profound effects on cell physiology (30, 42, 68, 69). Hence, we include the caveat that the tumor cell “seeds” are motile and actively remodel the microenvironment “soil” in concert with stromal and immune cells to continue to “fertilize” the soil by secreting and assembling ECM components and other cytokines, altering both the physical and chemical properties of the tissue to successfully colonize organs.

In ecology, restoring the niche of a specific species has been a successful way to increase the population; thus, restoring the environment to that of normal tissue may be a good therapeutic strategy (37). We may exploit a specific signaling pathway, localize key proteins, or transplant cells that can reconstruct a niche to help the reconstruction of a normal microenvironment and suppress the activation of DTCs. Restoring the niche can normalize malignancy so that cancer cells will either enter quiescence or become phenotypically normal (31, 70–76). Specifically, teratocarcinoma cells revert to a normal phenotype when implanted into a normal mouse blastocyst (77). Additionally, modifying ECM inputs in a 3D hydrogel culture can normalize genotypically malignant cells (75, 78). Similar observations have been made in plant tumors, where crown-gall teratoma cells can generate normal shoots following grafting and successive healthy tobacco plants (79). Targeting the abnormal microenvironment or restoring or engineering the microenvironment could successfully supplement current cancer therapies by suppressing the development of malignant phenotypes or even reversing tumorigenesis (12, 76, 80).

## Engineering the Biotic Microenvironment – Defining the ECM Niche

Three-dimensional (3D) biomimetic platforms allow for recapitulation of features of tissue architecture such as 3D cell–cell interactions, ECM composition and architecture, achieving *in vivo*- like mechanical properties and the diffusion profiles of signaling molecules (81–83). The simplest 3D culture model consists of single cancer cells or spheroids embedded in a natural matrix or cell-derived matrices of one ECM protein or hybrid of many proteins such as collagen type I, laminin-derived hydrogels, or alginate (44, 45, 84–86). Matrix porosity, fiber structure, and stiffness may be tuned by controlling polymerization conditions (85, 87, 88). Within such 3D models, cells rapidly proliferate and form spheroids or aggregates that recapitulate *in vivo*-observed tumor ECM formation, tumor cell–cell interactions, tumor-like molecular diffusion gradients, chemoresistance, and invasive metastasis characteristic of tumor progression (89–94). Using these naturally derived matrix mimetics, a dynamic range of stiffness can be realized usually in the range of ~100–1 MPa which allows for a closer approximation of *in vivo* mechanical cues (95, 96). However, due to the batch-to-batch variations of gels it is difficult to achieve consistency (32). Synthetic hydrogels derived from materials such as polyethylene glycol, self-assembling peptides, and poly (d, l-lactide-co-glycolide) offer the advantage of spatially defined environments with controllable physical parameters (97–99). Specific adhesion sites such as RGD-binding motifs can be incorporated into a hydrogel to model cell adhesion (97), and the controlled release of tumorigenic factors into hydrogels can shed light on specific aspects of tumor development (100). These engineered environments provide tractable platforms to dissect the role of external biochemical and mechanical stimuli on cell migration in 3D, mechanisms of cellular differentiation, gene expression, and cellular responses (83). Mixed materials featuring both natural and synthetic components offer the advantage of independently tuning variables such as mechanical stiffness, adhesion, peptide density, and matrix hydrophilicity (101).

Tissue is not homogeneous *in vivo* but has distinct topographies, as evidenced by differences in the architecture of fibrillar collagens and vasculature (48). In addition to stiffness, other physical properties such as dimension and topography have profound effects on cell fate and malignancy (30, 47, 48). Therefore, it is desirable to consider spatial heterogeneity in these artificial biological landscapes to incorporate contributions from both tissue stiffness and those due to topography. Laminin hydrogels form amorphous gels with no fibrils or spatial heterogeneities on the same scale as that of cells. To dynamically tune the protein concentration of these hydrogels, the proteins are commonly cross-linked. But matrix rigidity inevitably, and sometimes undesirably, increases with protein concentration (63). A key goal of developing biomimetic matrices currently underway is to develop a methodology that allows for independent control of material parameters such as protein distribution and alignment, and other physical properties (48).

## Defining the Biotic Cellular Components within the Tumor Ecosystem: Tumor–Stromal Interactions

Tissue homeostasis and architectural integrity are facilitated by the dynamic interactions between normal epithelial cells and stromal cells. Cancer cells alter the normal tissue landscape in efforts to transform the tissue microenvironment into one that is tumor-permissive (34, 35, 42, 51). Within tissue, heterotypic cell interactions between tumor cells and stromal cells can either drive or mitigate tumor development and metastatic potential (102–106).

Immune cells such as Tie2-expressing monocytes (107), mast cells (108), myeloid cells (109, 110), B cells (111), and macrophages (56) form cooperative relationships with malignant cells to create a tumor-promoting niche. By contrast, other immune cells such as natural killer cells (112) and dendritic cells (113) act as tumor-suppressive agents. Furthermore, these interactions are contextual; immune cells can either act as tumor-promoting or tumor-suppressive agents, as occurs with neutrophils (114) and regulatory T cells (115, 116). Immunotherapies using vaccines, immune adjuvants, cytokines, immune-modulating antibodies, and effector cells that exploit the immune response to cancer have been implemented, often in combination with radiation and chemotherapy (113, 117–121).

Similarly, other cells found in the reactive tumor stroma such as cancer-associated fibroblasts (CAFs), endothelial cells and pericytes can adopt either tumor-promoting or tumor-suppressive roles (122). CAFs have been found to promote tumor progression in breast and pancreatic cancers (104, 122–124). They support tumor proliferation by altering their metabolism (125, 126), increasing ECM production, and producing and digesting metabolites (113, 122). They also secrete growth factors such as VEGF, EGF, and TGF-a and chemokines including IL-1, IL-6, and CCLs (122, 123, 127) to recruit endothelial cells, pericytes, and inflammatory cells in building the tumor niche. These interactions promote the development of the tumor by encouraging proliferation, angiogenesis, inflammation, and metastasis. Due to their cooperative roles in the tumor microenvironment, CAFs have been targeted by various cancer treatments (128).

However, the angiogenic environment remains one of the most potent druggable targets (129) and one of the most successful stromal targets in cancer treatment (39, 130, 131). Angiogenesis or the sprouting of neovessels is induced early during the evolution of invasive carcinomas as observed in animal models and in humans (131). This process involves recruitment of sprouting vessels from existing blood vessels where endothelial cells proliferate, migrate, and organize functional tubular structures. Not only is the generation of neovasculature important to sustain proliferation of macroscopic tumors, it may play a functional role in tumor etiology to facilitate the transition from premalignant neoplasia to full-blown malignancy (131). The nascent angiogenic environment remains an attractive target in benign lesions in efforts to prevent full-blown malignancy.

Recent studies have begun to implicate lesser-interrogated relationships between tumor cells and other cell types within the microenvironment such as adipose cells and neuronal cells (132). Cancer-associated adipocytes (CAAs) promote tumor development and progression (133) by promoting tumor cell migration and invasiveness (134, 135). The molecular mechanisms underlying adipocyte-cancer cell communication are possible targets for cancer diagnosis and treatment, and could help to reveal the relationship between obesity-related metabolic disorders and cancer (136). Neuronal (nerve) cells have also been implicated in promoting malignancy (137). Specifically, these cells secrete factors that facilitate increased tumor proliferation. In turn, tumor cells secrete neurotropic factors and exon guidance molecules that induce sprouting of sensory nerves within the bone to increase neurite formation (138, 139). Consequently, patients experience cancer-related bone pain which can be relieved by the inhibition of nerve growth factor (NGF) (139). Additional characterization of the neurotransmitters or neuropeptides that mediate the cooperation between cancer and neural cells could reveal potential targets for cancer treatment.

## Building the Ecosystem – A Co-Culture Architecture in 3D

3D heterotypic platforms afford the flexibility of incorporating multiple cell types such as the co-culture of tumor cells and fibroblasts, macrophages adipocytes, and osteoblasts and endothelial cells (79, 140–143). These *in vitro* platforms simulate the *in vivo* network of interactions within the tumor ecosystem. From this point of view, when developing a co-culture system, one should consider (i) cell types and model complexity; (ii) the desired interactions between cell types; and (iii) population-level characteristics such as culture volume, cell density, population sizes, and the interactions between cell populations. These co-culture systems can be as simple as a sandwich model where one cell type is cultured on a confluent layer of a different cell type. This bilayer geometry can also be achieved using commercially available transwell culture systems and microfluidic platforms (144–148). A second commonly used geometrical arrangement involves the culture of multicellular tumor spheroids co-cultured with cells embedded in the surrounding surrogate matrix. These spheroids can be generated due to spontaneous aggregation, liquid overlay cultures, or using gyratory or spinner flasks or expansion from a single cell (149). These models have been used to study tumor progression and aid in the development of drug screening platforms (150). Spheroids generated from tumor or tissue biopsies may facilitate the development of individualized and patient-specific therapy (151). Additional complexity such as topography of interaction, degree of contact, and the number of cells that can be co-cultured can be achieved using a combination of microfluidics and micropatterning. Specifically, cell types that require different environments to maintain physiologically relevant signaling can be co-cultured in microfluidic channels to facilitate exchange of molecules without direct contact. Using this platform, their respective environments are separated by semi-permeable membranes or gels within microfluidic channels (152, 153) where their growth media are separated using laminar flow, or grown in semi-connected liquid compartments (154). To date, the majority of co-culture studies have involved two populations such as normal cells and malignant cells (155). However, many different types of cells are involved in the process of tumorigenesis, which intrinsically increases the complexity. The combination of microfluidics and 3D microfabrication of ECM scaffolds has been used to generate self-assembly of *in vitro* “organs on a chip” (156) such as the brain, liver, and gut (157–159). Interactions among more than two cell types have been studied in a defined synthetic ecology for bacteria (160), or in model systems of organs such as the endothelium (161). Thus, a future application to recapitulate these interactions might be achieved by incorporating tumor cells in these organotypic models.

## Abiotic Components within the Tumor Ecosystem: Oxygen, pH, Stiffness, and Chemokine Gradients

The acellular microenvironment of a tumor is characterized by cells and biochemical components and by their complex interactions with the physical and chemical parameters such as stiffness, pH, oxygen tension, interstitial pressure, and fluid flux (42, 162–164). Both tumor and stromal cells create gradients of secreted cytokines and growth factors, which contribute to altered proliferation and directed cell migration to facilitate tumor progression, dissemination, and invasion (57, 165, 166).

As tumors proliferate, their energy and oxygen requirements often cannot be met by existing tissue vasculature. Hypoxia is also associated with drug resistance as low oxygen tensions affect the cell cycle and slow cycling cells are thought to be minimally affected by treatments targeting the cell cycle (167). It has also been implicated in upregulation of genes that regulate cell proliferation, ECM production, cell adhesion, and cell invasion through induction of the hypoxia-inducible factor (HIF) family of transcription factors. Neoangiogenesis in part alleviates growth-induced hypoxia but tumor vasculature lacks the normal hierarchical arrangement of artery–arteriole–capillary, resulting in intercellular gaps that leak fluids, blood, and fibrins, and inefficient oxygen delivery (168). Consequently, within the growing mass, there are regions of hypoxia where certain cancer cells are deprived of oxygen (169). In response, tumor cells may undergo a metabolic shift causing acidosis (170, 171). Hydrogen (H^+^) ions generated by tumor cells during both aerobic and anaerobic glycolysis, glutaminolysis, and ATP hydrolysis are transported to the extracellular milieu (172, 173) creating an acidic tumor microenvironment (174). These acidic environments are thought to be permissive for tumor invasion and successive metastasis by inducing cell death in the surrounding normal tissue and degradation of the ECM.

In addition to the chemical variations, a myriad of physical attributes of the tumor microenvironment also play important roles by imparting mechanical cues that promote the malignant transition of cancer cells and increased metastasis (30, 42, 175, 176). Cells encounter multiple mechanical cues at each step of the metastatic cascade. They traverse complex ECM arrangements and topographies during invasion into surrounding tissue and in the vascular system during intravasation and extravasation (30). These dynamic mechanical inputs are derived from changes in stiffness, fibril structural architecture, and shear stress. Shear flow directly affects extravasation by modulating adhesion at blood vessel interface (30). Stiffer areas of fibrosis with increased ECM deposition are found around some invasive ductal carcinomas and metastatic lesions residing at lymph nodes and bones (177). In these patients, a higher risk of developing bone and lymph node metastasis, and disease recurrence was observed (177). Tumor dormancy is modulated by the microenvironment. Cells remain quiescent in initially non-permissive microenvironments, but transition to a proliferative state based on changes in the ECM composition and stiffness (12, 178).

## Building the Ecosystem-Manipulating Abiotic Conditions

**Figure 1:**
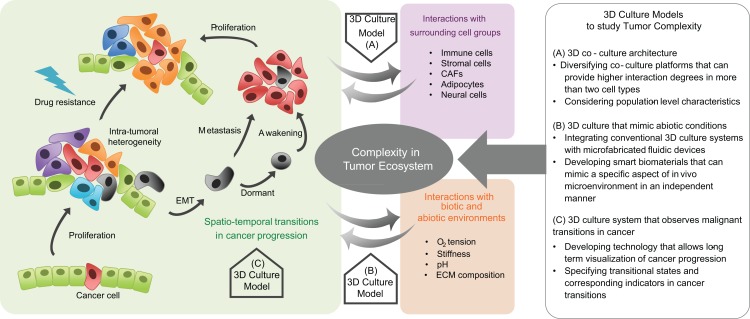
**Complexity in tumor ecosystem and 3D culture models**. Cancer is a complex disease in which the cancer cell population dynamically evolves and the diversity of heterotypic interactions between cancer cells, surrounding cells, and environmental factors is spatiotemporally regulated. Therefore, preclinical models that incorporate factors that play critical roles in the dynamic tumor progression, within a defined biomimetic landscape are needed. Three-dimensional culture models help to deconstruct the complexity of cancer. Model systems can be engineered to recapitulate tumor cell-surrounding cell interactions, the physicochemical characteristics in an abiotic tumor environment, and the malignant transitions in tumor progression. They can provide insight into the evolutionary and ecological aspects of tumor progression, with relevant therapeutic implications.

3D culture models have been used to bring *in vivo* abiotic conditions to cultured tumor cells, with effects such as hypoxia and acidosis on tumorigenicity (179, 180). Using these platforms, angiogenesis (181–185), cell migration (166, 186, 187), invasion (188), metastasis (189), and epithelial–mesenchymal transition have also been examined (190). They have also provided a means to study phenotypic-based pharmaceutical efficacy to test the likelihood of acquisition of drug resistance (191–195). Specifically, cells grown under hypoxic conditions in 3D culture models show higher resistance to toxins than the same cells when cultured in 2D grown on plastic (196–199). Microfluidic chips fabricated with soft-lithography methods offer the possibility of simultaneously reproducing both physical and chemical abiotic components within tissue mimetics (166, 200–204). Defined geometries allow confined cell growth or cell–cell contact, with fluid flow controlled to generate physiological shear stresses or chemical gradients, with serial fluid exchange performed to deliver nutrients or chemicals on defined time-tables, or with integrated mechanical components able to exert periodic stresses on cells or the ECM (156, 191, 205–207). By combining these fabrication techniques, the organ-on-a-chip was developed, which recapitulates a broad range of *in vivo* physiological functions for organs such as the brain, liver, and gut (208–210). These devices also permit the analysis of tissue-specific responses in reaction to external physical stimuli such as stiffness or shear stress and biochemical perturbations such as drugs or toxins (211–213). A recent application in which dynamic hydraulic compression was applied to human bone marrow and adipocyte-derived stem-cells-on-chips resulted in increased bone differentiation as measured by osteogenic gene expression and production of bone-specific ECM components (214). These principles can be applied to cancer studies (215) in which *in vitro* 3D models of lymphoma, pancreatic, breast, prostate, and oral cancers have already produced clinically relevant results (192). These and the aformentioned model systems are summarized in Figure 1.

## Malignant Transitions in the Tumor Ecosystem

The ecological perspective on cancer introduced so far has been based primarily on studies on many genetic and molecular factors that initiate, drive, and promote cancer. Even though many causative elements are involved in malignant progression, the emergent malignant properties such as drug resistance and metastatic potential are still poorly predicted and managed, because our knowledge about the population-level dynamics of cancer is limited (216–219). Many phenotypic transitions in cancer cell populations occur during tumor progression: transitions from a single cell to multiple cells; from slow to fast growth; from proliferation to migration; from oxidative phosphorylation to anaerobic glycolysis; from epithelial to mesenchymal phenotype; from drug-sensitive to drug-resistant; and from dormant to metastatic (220–223). Understanding these transitions in the context of population dynamics might open new avenues of cancer treatment, as preventing the transitions toward malignancy could make a tumor manageable and treatable by hampering its adaptive progression. Using these microfluidic platforms in which both the mechanical inputs and chemical gradients are controlled may yet simulate *in vivo* evolution of tumor heterogeneity and phenotypic plasticity due to environmental perturbations.

## Visualizing Malignant Transitions in the Tumor Ecosystem

Imaging modalities such as magnetic resonance imaging (MRI), computed tomography (CT), positron emission tomography (PET), and microscopy offer the advantage of long-term monitoring in human patients with lesions can be imaged over a period of time (224). In particular, MRI and PET also detect changes in tumor metabolism and pH (174, 225). However, the use of such imaging methods to monitor human disease is faced with several drawbacks (226–229). Namely, disease can be detected only after several rounds of tumor cell proliferation, and single cell dynamics cannot be readily achieved (224). Ideally, diagnosis should occur at the earliest stage of malignant transition and/or single cell resolution if efforts are to be successful for therapeutic intervention. Murine models remain an attractive option to examine tumor etiology and progression as one can interrogate human cell lines using immune-compromised strains and similarly examine syngeneic cancer cell lines for each strain (230). But these preclinical models share the same imaging bottlenecks as observed in humans. The major limiting factor when imaging organs is that biological tissue is opaque and scatters light such that image quality deteriorates rapidly if one attempts to image deeper into thicker samples (231). Conventional confocal microscopy (one and two photon) cannot image deeper than a few hundred microns within organs that span millimeters to centimeters (232). Combining adaptive optics with two-photon fluorescent microscopy increases the achievable spatial resolution to penetration depths of ~1mm (233). Intravital microscopy using principles based on two-photon microscopy offers the flexibility of imaging single cell dynamics within thick tissue (234–236). However, longitudinal studies in murine models often require invasive techniques such as an imaging window and fluorescent reporters to visualize single and collective tumor cell dynamics (237). These windows often limit the area of interrogation. Hence, the sacrifice of many mice is needed to achieve acceptable statistics. In addition, these techniques require specific expertise, and are currently low throughput.

Combining imaging modalities and *in vitro* 3D culture models that recapitulate physiologically relevant aspects of tumor progression and metastatic disease as an alternative ­pre-clinical model allows us to image single cell dynamics (238). Using engineered platforms, single tumor cell intravasation and tumor cell proliferation/motility in different microenvironments have been visualized (239–243). These examples hint at the power of combining imaging modalities with 3D *in vitro* platforms to study heterotypic cell interactions, evolution of tumor heterogeneity, and acquisition of drug resistance. However, it would be advantageous if we could merge technologies that allow for visualization and quantitation of physical properties such as ECM stiffness and changes in topography of the tissue. In this section, we present some ideas for future consideration. One area involves monitoring the dynamic mechanical measurements of the ECM throughout the entire process of tumorigenesis. Several methods for determining physical properties of surrogate ECM gels and cells exist such as atomic force microscopy, which can be used to mechanically probe thin gels and superficially in excised tissues (~nm), and traction form microscopy, which can resolve forces within thick hydrogels where the physical properties are known (244–246). While these techniques are powerful, a technique that can probe the mechanical properties at depths >100 μm with sub micron resolution in thick samples eventually extending to an animal model without *a priori* knowledge of the physical properties of the tissue is desirable. Optical traps have been used to characterize gels and other materials (247), and recently have even shown the ability to characterize the viscoelastic properties of living cells (248). The combination of imaging the local structure and components of the ECM while simultaneously measuring its mechanical properties will reveal correlations between the two, and how they change with time as cells remodel and react to their environment. Combining light microscopy and an optical trap platform may be one method to simultaneously visualize and measure the changes in the physical properties of the local microenvironment in 3D *in vitro* platforms.

## Estimating the Risk of Emergent Malignancy in Cancer

One way of modeling tumor growth is by a logistic growth curve in which growth is initially exponential but then stabilizes (249). Recently a review proposed consider an alternative approach from evolutionary dynamics, where an allee effect may be explored for therapeutics. Briefly, the Allee effect is defined as a decline in individual fitness at low population size or density. A population with a strong allee effect is one that is stable at intermediate numbers but may become extinct at numbers that are either too small or too large (249). Therefore, estimating the risk of such a transition in a cancer population may predict the likelihood of progression, and also provide opportunities to prevent population diversification, metastasis, and drug resistance. One possible application is to eradicate quiescent DTCs that are able to enter dormancy and evade targeted/conventional therapies (250). These cells are thought to be rare and few members of the original primary tumor cells (250). Discovering the dormancy mechanism of DTCs has been the subject of numerous studies, as it is considered an important mechanism of tumor recurrence that is responsible for a large proportion of tumor-related deaths (251). Understanding the microenvironmental regulation of producing a strong Allee effect on these rare cells might also help to advance current therapies by exploiting a rapid collapse of this tumor sub-population. In the field of ecology, a critical transition is an abrupt and unexpected change of a complex system, such as a sudden climate change with rapid extinction of a species (252). In homogeneous and highly connected networks, local losses due to external stresses can be normalized by subsidiary inputs from linked units in the network; however, when the amount of stress exceeds a certain critical level, the system rapidly collapses (253, 254). If we treat heterotypic cell interactions, as those observed for interspecies interactions, and tissue architecture likened to spatial organization of species, then tumors are similar to ecologically complex systems. It is possible that a critical transition where tumor cells become extinct or normalized may be induced by normalizing tissue architecture or eradicating supportive tumor–stromal interactions (249).

Unfortunately, there have been few empirical results demonstrating sudden systematic transitions in cancer cell populations. Several 3D models that recapitulate the process of epithelial–mesenchymal transition in cancer have been observed at the single spheroid level (255–257). However, theoretical studies on population level changes have supplemented these experimental observations (249, 258, 259).

In conclusion, studying ecological aspects of cancer provides us with the tools to understand cancer complexity, and the power to prevent further progression toward emergent malignancy. Direct visualization of tissue dynamics has refined our understanding of the basic principles of cell migration, lineage commitment, and the establishment of tissue architecture. Future studies in which 3D culture models are coupled with microfluidics and appropriate imaging modalities will begin to examine the singular inputs and interplay of biotic and abiotic components within tumor ecosystems. We may yet identify universal signals produced when a tumor approaches a malignant transition, such as acquisition of drug resistance, dormancy, or metastasis, to enable better management of cancer.

Transforming these ecological inputs into meaningful information for patient care will be challenging. Evolutionary and ecological theories may be the unifiers in our understanding of cancer. Advances in phylogenetic reconstruction and agent-based modeling will guide our understanding of somatic evolutionary pathways (5, 260, 261). In addition, mathematical models that incorporate tumor heterogeneity and the microenvironment allow for derivation of potential outcomes as tumor cells adapt to abiotic changes, such as hypoxia and acidosis, and to chemotherapy (20, 262–264). Models integrating observations and experimental data will continue to refine our understanding of the adaptive tumor landscape (263–266). Advanced 3D model systems will provide tools to interpret this complexity, which could lead to an alternative description of emergent cancer progression.

## Conflict of Interest Statement

The authors declare that the research was conducted in the absence of any commercial or financial relationships that could be construed as a potential conflict of interest.
